# Care-seeking at patent and proprietary medicine vendors in Nigeria

**DOI:** 10.1186/s12913-015-0895-z

**Published:** 2015-06-12

**Authors:** Lisa M. Prach, Emily Treleaven, Chinwoke Isiguzo, Jenny Liu

**Affiliations:** Global Health Sciences, University of California, San Francisco, 550 16th Street, Mission Hall: Global Health & Clinical Sciences Building, San Francisco, CA 94158 USA; Society for Family Health, No 8 Port Harcourt Cresent, Area 11 Garki, Abuja, Nigeria; Department of Social and Behavioral Sciences, University of California, 3333 California St., Suite 455, San Francisco, CA 94143 USA

**Keywords:** Care-seeking behavior, Customer interaction, Drug shops, Fever, Headache, Patent and proprietary medicine vendors, Nigeria

## Abstract

**Background:**

To achieve health development goals, policymakers are increasingly focused on improving primary care in low- and middle-income countries, and private sector drug retailers offer one channel through which basic services may be delivered. In Nigeria, patent and proprietary medicine vendors (PPMVs) serve as a main source of medications, but little is known about their clientele or how care is sought at PPMVs for common illnesses. We explore differences in care-seeking at PPMV shops based on the most commonly reported symptoms.

**Methods:**

In Kogi and Kwara states, Nigeria, 250 PPMV shop workers and 2,359 customers purchasing drugs were surveyed, and each worker-customer interaction was observed. Multivariate regression analysis was used to assess the association of commonly reported symptoms with care-seeking behavior prior to attending the shop and while interacting with the provider at the shop.

**Results:**

Most customers sought care for headache (30.5 %), fever (22.9 %), cough/cold (18.1 %), or diarrhea (8.4 %). Customers with fever were more likely to report being diagnosed by a formally trained person, to have discussed the illness with and be examined by the shop worker, and have more difficulty paying. In contrast, customers with headache symptoms were less likely to experience these outcomes and spent less money purchasing drugs. Those reporting cough or cold symptoms were less likely to have been diagnosed by a formally trained person, waited longer before visiting the PPMV shop, and were more likely to discuss the illness with the shop worker, but were less likely to be examined or to recommend the purchased drug themselves. If a sick child was brought to the shop, a discussion of the illness and an exam were more likely and more money was spent on drugs.

**Conclusions:**

Because care-seeking behaviors vary by symptoms and the sick person’s age, PPMVs should be trained to treat common illnesses for which customers are unlikely to seek a formal medical consultation. Interventions aimed at improving primary care need to target the places where most people access care, and equip PPMV workers with knowledge and tools to provide basic services.

## Background

In order to achieve universal health coverage, expanded access to basic primary care services is needed, especially in low- and middle-income (LMIC) countries [1]. Although access to care and child survival have improved over the last three decades [2, 3], many people in LMICs continue to face a variety of structural and social barriers to obtaining formal primary care services [4], resulting in inequitable access [1].

Across many countries in Sub-Saharan Africa (SSA), a large portion of care is delivered through private sector providers, ranging from traditional healers and informal providers to formally trained nurses, midwives, and clinicians [5]. In many instances, the point of first care-seeking occurs at drug shops whose workers may not necessarily be formally trained in healthcare provision, and thus often act as informal providers. For example, private drug shops are the first place where treatment is sought for common childhood illnesses for between 15 % and 83 % of episodes in different SSA countries [6]. Given their numbers, market share, and presence in rural communities, drug shops represent an important opportunity for improving primary health care, and several SSA countries have included drug retailers in national health interventions [7–9].

In Nigeria, patent and proprietary medicine vendors (PPMVs), defined as “a person without formal training in pharmacy who sells orthodox pharmaceutical products on a retail basis for profit” [10], provide the main source of medicine for many common illnesses. The estimated 200,000 PPMVs operating in Nigeria [11] are the first source of care for up to 55 % of under-five child illnesses, such as malaria and diarrhea, and 35 % to 55 % of adult malaria treatment [12–16]. In addition to selling drugs, PPMVs can be a source of advice about illness and drug therapy [17] in place of more formal care at health facilities, even if they are relatively accessible.

Legally, PPMVs are permitted to sell a limited number of pre-packaged, over-the-counter medicines, but are prohibited from selling prescription medications (e.g. antibiotics) or conducting invasive medical procedures (e.g. malaria rapid diagnostic tests) [18]. Formal medical or pharmacy training is not required for PPMV licensure [17], but by convention, the minimum educational attainment has been primary schooling [10] and most complete an apprenticeship with a more senior PPMV before opening their own shop [19, 20]. Because the primary function of a PPMV shop is retail sales and not healthcare, customer demand often dictates the products and services provided [10], and may lead to PPMVs practicing beyond their legal scope [21–24].

A recent systematic review of PPMV practices shows that PPMVs provide medicines and services for a wide variety of health needs, including malaria, respiratory infections, diarrhea, common cough and cold, tuberculosis, and reproductive health; however, the quality of these services is low [25]. PPMVs generally have low health knowledge and poor health treatment practices, stock poor quality medicines (e.g. partial or repackaged doses [26], and sub-standard formulations [27, 28]) as well as medicines and commodities they are prohibited from selling [21–24]. While the literature is dominated by studies of malaria, the available evidence indicates that PPMVs’ limited knowledge of proper treatment practices contributes to sales of inappropriate, ineffective, and incorrect drugs for many health problems. The review also highlighted the lack of information about the full range of services offered at PPMVs as most studies focused on a single health focus; with a few notable exceptions, little is known about the spectrum of conditions that customers seek treatment for, how PPMVs interact with customers in order to dispense medications for those conditions [10, 21], and the variety of drugs sold at PPMV shops.

In order to fill these gaps, the aim of this paper is to explore differences in care-seeking behavior based on the type of symptoms most commonly reported (fever, headache, diarrhea, or cough/cold) by PPMV shop customers. We investigate two sets of outcomes: (1) care-seeking behavior in terms of getting diagnosed by a formally trained person prior to attending the PPMV shop, and time waited before attending the PPMV shop; and (2) PPMV-customer interactions based on whether or not the illness was discussed, conduct of an exam on the sick person, who recommended the drug dispensed, cost of treatment, and self-reported difficulty in paying. Findings are discussed in light of policy considerations that seek to improve the quality of primary care services by PPMVs for the most common health conditions.

## Methods

### Study area and sample selection

The study was conducted in Kogi and Kwara states in the North Central geopolitical zone of Nigeria. Kogi State, located in central Nigeria just south of the Federal Capital Territory and comprised of 21 local government areas (LGAs) over 30,355 km^2^, houses about 4.0 million people from a diverse ethnic background, among which the Igala and Ebira tribes have the largest representation [29]. Kwara State, lying to the east of Kogi, is more sparsely populated with about 2.4 million people residing in 15 LGAs over 32,500 km^2^ [30]. The principal tribal groups are the Yoruba, but Nupe, Bariba and Fulani are also present. In both states, agriculture forms the basis of the economy (e.g. cassava, yams, rice, cotton, coffee), but mining activities also contribute. At US$1,386 and US$1,585, Kogi and Kwara rank 24^th^ and 20^th^, respectively, among Nigeria’s 36 states and the Federal Capital Territory in terms of per capita gross domestic product [31].

From April to September 2013, all PPMV retail establishments selling drugs in the study states were enumerated. From a complete listing of 2,083 shops (1,088 in Kogi, 995 in Kwara), 250 were selected (125 per state) for this study through a modified, stratified random sampling approach: due to security concerns and logistical challenges, the sampling of PPMV shops was centered around four focal urban or peri-urban hubs in each state (Kogi: Ayangba, Ida, Lokoja, and Okene; Kwara: Ajase-Ipo, Bode-Sadu, Ilorin, and Omu-Aran). Shops within a 25-km radius (the distance that a surveyor could safely and feasibly travel to and from a central post in one day in each hub, chosen in consultation with local officials) from the hub center were randomly selected, stratified across urban, peri-urban, and rural locations.

All PPMV shops chosen in the sampling process from the census were eligible to participate. From Monday to Saturday, each surveyor (two if multiple local language capabilities were needed) visited one shop per day, arriving at opening hours and leaving when the shop closed at night. If a shop could not be located (due to absent provider, closure, or incorrect address), a shop in the vicinity that self-identified as a PPMV and was not chosen in the original sample was randomly selected as a replacement (30 in Kogi, 24 in Kwara). Shop workers were not given advance notice of a visit. Upon arrival at the shop, written informed consent was obtained from the person in charge at the time (79.2 % owner, 6.8 % manager, 13.2 % apprentice, 0.8 % other). All shops approached for consent agreed to participate.

### Data collection

Two data collection activities took place at each shop: (1) a shop survey and (2) customer observations and exit interviews. Shop surveys were administered at the beginning of the day when customer traffic was lower, but interruptions for attending to customers were allowed. The shop survey captured information about the shop owner’s and workers’ sociodemographic characteristics, knowledge of and self-reported practices for treating common childhood illnesses (malaria, diarrhea, and pneumonia), social and institutional resources for drug and healthcare advice, and types of drugs stocked. At the end of the day, the shop worker was given a small gift—a wall clock valued at 500 Naira (~US$3.13)—as compensation for participation.

Throughout the day, surveyors recorded observations for all customers coming to the shop according to a standardized form. These included time of visit, waiting time, with whom the customer interacted, and topics of discussion (e.g. history of illness, symptoms experienced, drugs needed to treat the illness, prevention information, referral), whether any examination (physical or otherwise) was conducted, and whether any drugs were purchased. Surveyors did not engage with either the shop worker or the customer during an interaction. If customers purchased a drug, s/he was approached after leaving the shop to obtain verbal informed consent, verify eligibility, and be administered the exit survey. Only customers over age 12 were eligible, the minimum age for giving informed consent commensurate with the study’s minimal risk level as determined in consultation with local officials and approved by the local Institutional Review Board. The exit interview captured the sociodemographic characteristics of the customer and the sick person, symptoms experienced and being treated, source of illness recognition, types of drugs purchased, and amount spent. On average, customer observations took less than five minutes and exit interviews about four minutes. During times of high customer volume, some customers were missed and surveyors recorded the number of customers missed while they were occupied with an exit interview. No personally identifying information for customers was recorded and no compensation was given. Among 5,123 customer observations recorded, 3,014 (59.1 %) purchased a drug, of whom, 2,442 (81.0 %) completed an exit interview.

### Care-seeking behavior

Customer care-seeking behavior was operationalized as two measures: (1) having been diagnosed by a formally trained person and (2) time waited before visiting the PPMV shop since the onset of symptoms. Customers are coded as being diagnosed by a formally trained person if they responded to the question, “Who figured out what illness this is?” with responses of doctor/nurse, pharmacist, or community health extension worker (CHEW); responses of myself/relative/friend, chemist/PPMV, traditional healer, or lab technician were coded as not being diagnosed by a formally trained person. Time waited before attending the PPMV shop was self-reported by the customer and coded as a binary indicator (0-1 day vs. 2 or more days); additional ways of coding of this variable (i.e. continuous, ordered count) produced similar results.

### Shop interaction and drug purchase outcomes

Shop interactions were characterized with three binary variables. (1) Whether or not the customer and PPMV discussed the illness was based on observation, coded 1 if the symptoms or recent history of illness were mentioned by either the shop worker or the customer during the interaction. (2) Whether or not the sick person was physically examined was determined by the surveyor if s/he observed that any conduct of an “exam” occurred, which included looking at physical appearance, inspecting parts of the body, measuring blood pressure or temperature, or checking eye color. (3) Whether or not the customer, a family member, or a friend recommended the drug purchased was based on answers to the question, “Who mainly recommended the drugs that you purchased today?” and coded as 1 if the response was “myself” or “family/friends” and 0 for responses of “doctor/nurse”, “pharmacist”, “chemist/PPMV”, “CHEW”, or “traditional healer”.

Two cost-related outcomes were constructed: (1) total cost in Naira and (2) whether or not the customer had problems paying. In the exit interview, customers were asked, “How much did all of this cost?” for the drugs purchased (excluding any other commodities). A follow-up question probed about whether or not s/he had problems paying for the drugs purchased.

### Data analysis

Multivariate analyses were performed using logistic regression for all outcomes except for drug cost, which was log-transformed and analyzed using linear regression. Standard errors were clustered by drug shop to account for autocorrelation among customers attending the same shop. Covariates for PPMV shop worker and customer characteristics were selected based on *a priori* hypotheses of determinants of care-seeking. Shop worker characteristics included age (years), gender, formal medical training (coded as 1 if self-reported as a pharmacist, nurse, midwife, doctor, or CHEW and 0 if self-reported as a current apprentice, completed apprentice, lab technician, or none), reported offering tests or exams (e.g. taking temperature with a thermometer, measuring blood pressure, checking eyes, or performing a physical exam), location (urban, peri-urban, or rural), and state. Customer characteristics included gender, age (i.e. age 13-18, 19-25, 26-40, or 41 and over), highest education completed (primary, secondary, or more than secondary), tribe, living in the community where the PPMV shop is located, having a clinic near his/her home (allowing the customer to self-define “near”), age of the sick person (i.e. under age 5, 5-17, or 18 and over), if the sick person is a child (i.e. age 18 or under) and male, if the sick person is a child and present at the PPMV shop, and the most commonly self-reported symptoms (i.e. fever, diarrhea, headache, or cough/cold including congestion, cough, cold, or sore throat). Symptoms reported in low frequencies (e.g. sweating, nausea/vomiting, shortness of breath, body aches/joint pain, dizziness/lightheaded, dark urine, or chills/shivering) were not included as they did not logically aggregate into larger categories. Wealth index quintiles were constructed using standard principal components analysis of household asset indicators [32] and were included in all multivariate analyses.

Interactions were explored to investigate differential effects according to (1) the degree of concordance between the PPMV and the customers’ tribal affiliation; (2) gender with the age of the sick child; (3) wealth status with the gender of the sick child; (4) wealth status with source of diagnosis; and (5) the experience of fever symptoms with age of the sick child. None of these interactions were significant and were excluded in the final analyses.

The type of drug purchased (e.g. ACT, other antimalarial, antibiotic, anti-diarrheal, analgesic, or vitamin/supplements) per customer was descriptively analyzed according to reported symptoms. P-values were considered significant if *p* < 0.05. Analysis was performed using StataSE version 13.1.

### Ethical considerations

The Nigerian Health Research Ethical Review Committee approved this study. Verbal informed consent was obtained from customers and written informed consent from shop workers. Funding sponsors did not have any role in the study design, execution, or publication.

## Results

### Customer characteristics

A total of 2,359 customers over age 12 agreed to complete an exit interview after purchasing a drug from a sampled shop. About half (52.3 %) were male and 15.8 % were children (age 13-18; see Table 1). Nearly half (46.7 %) had completed secondary education. While customers came from several tribes, Yoruba (39.0 %) and Igala (25.2 %) were most highly represented. Nearly 85 % of the customers lived in the same community as the PPMV shop and over 60 % reported having a clinic near their home. Most customers were purchasing drugs for a sick adult (74.6 %), including for him/herself or for someone else over 18. Among sick children (age 18 or under), 33 % were present at the shop (equivalent to 8.4 % of the total sample). More customers self-reported headache (30.5 %) than any other symptom, followed by fever (22.9 %), cough/cold (18.1 %), and diarrhea (8.4 %).

**Table 1:** Sample characteristics

Customer characteristics (N=2359)		Number	Percent
Symptoms reported	Fever	539	0.229
	Diarrhea	199	0.084
	Headache	719	0.305
	Cough/cold	427	0.181
Male		1228	0.523
Age	13-18	372	0.158
	19-25	592	0.251
	26-40	901	0.382
	41+	494	0.209
Educational attainment	Primary	762	0.323
	Secondary	1101	0.467
	Higher	496	0.210
Tribe	Ebira	240	0.103
	Fulani	205	0.088
	Igala	589	0.252
	Yoruba	910	0.390
	Other	390	0.167
Wealth quintile	Poorest	487	0.207
	Second	457	0.195
	Third	468	0.199
	Fourth	468	0.199
	Richest	468	0.199
Lives in the community		1993	0.845
Has a clinic near home		1402	0.631
Sick person’s age	<5	293	0.124
	5-17	306	0.130
	18+	1760	0.746
Sick child (<18) is male		272	0.115
Sick child (<18) is present at PPMV	198	0.084	
PPMV characteristics (N=250)			
Age^a^		32.9 (11.5)	
Male		145	0.587
Has formal training^b^		49	0.198
Offers tests/exams		141	0.564
Location	Urban	70	0.280
	Peri-urban	85	0.340
	Rural	95	0.380
State	Kogi	124	0.496
	Kwara	126	0.504

The denominator for each characteristic excludes missing or unknown values

^a^Values shown as mean (standard deviation)

^b^Self-reported pharmacist, nurse, midwife, doctor or community health extension worker

### PPMV and shop characteristics

The mean age of the person in charge at sampled PPMV shops was 32.9 years, and more than half were male (58.7 %). Less than 20 % of shop respondents reported having any sort of formal medical training (i.e. doctor, nurse, midwife, pharmacist or CHEW), but over half (56.4 %) reported offering some type of test or exam.

### Care-seeking behavior

Table 2 displays the customer care-seeking behavior outcomes occurring prior to attending the PPMV shop. Overall, only 7.8 % of customers were diagnosed by a formally trained person. In multivariate analysis (see Table 3), customers reporting fever symptoms were more than twice as likely to have been diagnosed compared to customers not reporting fever (OR = 2.112; 95 % CI 1.428-3.123). In contrast, customers reporting headache or cough/cold symptoms were half as likely to have been diagnosed compared to those not reporting these symptoms (OR = 0.645; 95 % CI 0.427-0.974 and OR = 0.604; 95 % CI 0.366-0.997, respectively). Customers over age 40 (OR = 1.973; 95 % CI 1.112-3.500) or in the highest two wealth quintiles (OR = 2.073; 95 % CI 1.205-3.567 and OR = 2.017; 95 % CI 1.142-3.563) were significantly more likely to be diagnosed by a formally trained person. Customers with only primary schooling were less likely to be diagnosed before the shop visit (OR = 0.523; 95 % CI 0.284-0.964). Although only marginally significant (*p* < 0.10), there was a slight increase in likelihood of being diagnosed if the customer reported living near a clinic (OR = 1.558; 95 % CI 0.999-2.430).

**Table 2:** Care-seeking outcomes

Care-seeking behavior	N	%
Diagnosed by a formally trained person^a^	185	0.078
Number of days sick before visiting a PPMV shop		
0-1 days	811	0.344
2+ days	1548	0.656
Customer-PPMV interaction at the shop		
Customer and PPMV discussed illness	972	0.412
Sick person was examined	172	0.073
Customer/family/friend recommended drug^b^	1153	0.489
Drugs purchased		
Mean (SD) cost (Naira)		85.90 (3.13)
Mean (SD) cost by symptom reported		
Fever^c^		203.71 (2.73)
Diarrhea^d^		72.49 (3.04)
Headache^e^		69.11 (3.09)
Cough/cold^f^		111.37 (3.02)
Customer had problems paying^g^	233	0.102
N		2359

SD = standard deviation

^a^Customer responded to the question, “Who figured out what illness this is?” Formally trained personnel include doctor/nurse, pharmacist, or community health extension worker (CHEW) and exclude myself/relative/friend, lab technician, chemist/PPMV, and traditional healers

^b^Customer responded to question, “Who mainly recommended the drugs that you purchased today?” Excludes doctor/nurse, pharmacist, chemist, CHEW, and traditional healer

^c^ *N* = 537

^d^ *N* = 199

^e^ *N* = 717

^f^ *N* = 423

^g^ *N* = 2284

**Table 3:** Logistic regressions predicting the diagnosis by a formally trained person and time waited before visiting the PPMV (odds ratios)

	Diagnosed by a formally trained person		Waited 2+ days before visiting the PPMV	
	OR	95 % CI	OR	95 % CI
Symptoms reported				
Fever (ref: no fever)	2.112***	[1.428 - 3.123]	2.751***	[2.082 - 3.636]
Diarrhea (ref: no diarrhea)	0.620	[0.298 - 1.288]	0.925	[0.640 - 1.335]
Headache (ref: no headache)	0.645**	[0.427 - 0.974]	0.567***	[0.456 - 0.706]
Cough/cold (ref: no cough/cold)	0.604**	[0.366 - 0.997]	2.055***	[1.555 - 2.716]
Male (ref: female)	1.063	[0.734 - 1.541]	0.982	[0.800 - 1.205]
Age (ref: 19-25)				
13-18	0.720	[0.356 - 1.455]	0.815	[0.585 - 1.135]
26-40	1.343	[0.835 - 2.161]	1.004	[0.770 - 1.311]
41+	1.973**	[1.112 - 3.500]	1.092	[0.787 - 1.513]
Education (ref: secondary)				
Primary	0.523**	[0.284 - 0.964]	0.946	[0.711 - 1.259]
Higher	1.063	[0.681 - 1.658]	0.967	[0.724 - 1.292]
Tribe (ref: Ebira)				
Fulani	1.144	[0.334 - 3.916]	0.655	[0.344 - 1.249]
Igala	0.685	[0.345 - 1.364]	0.622**	[0.394 - 0.980]
Yoruba	0.528	[0.240 - 1.161]	0.805	[0.485 - 1.337]
Other	0.680	[0.344 - 1.346]	0.666*	[0.416 - 1.068]
Wealth (ref: third quintile)				
Poorest	0.421*	[0.177 - 1.005]	1.725***	[1.146 - 2.595]
Second	0.866	[0.468 - 1.602]	1.186	[0.856 - 1.643]
Fourth	2.073***	[1.205 - 3.567]	0.793	[0.583 - 1.080]
Richest	2.017**	[1.142 - 3.563]	1.149	[0.823 - 1.604]
Lives in community (ref: lives outside community)	0.724	[0.432 - 1.213]	1.027	[0.752 - 1.401]
Clinic near home (ref: no clinic near home)	1.558*	[0.999 - 2.430]	0.977	[0.772 - 1.236]
Sick person age (ref: 18+)				
<5	1.435	[0.780 - 2.640]	1.441*	[0.952 - 2.181]
6-17	0.658	[0.327 - 1.323]	0.944	[0.663 - 1.345]
Sick person is male <18 (ref: female or 18+)	1.242	[0.640 - 2.412]	1.132	[0.737 - 1.740]
Diagnosed by formally trained person (ref: not diagnosed by formally trained person)	3.556***	[2.213 - 5.715]		
Kogi (ref: Kwara)	1.113	[0.549 - 2.257]	1.162	[0.785 - 1.719]
Location (ref: urban)				
Peri-urban	1.384	[0.778 - 2.460]	0.972	[0.706 - 1.339]
Rural	1.392	[0.741 - 2.615]	1.324	[0.937 - 1.870]
N	2189		2189	

ref = reference

***p < 0.01, ** *p* < 0.05, * *p* < 0.10

Standard errors are clustered at the shop level. 95 % confidence intervals in brackets

More than 65 % of customers waited two or more days before visiting the PPMV shop (see Table 2). Symptoms that predicted waiting longer included fever and cough/cold symptoms (OR = 2.751; 95 % CI 2.082-3.636 and OR = 2.055; 95 % CI 1.555-2.716, respectively), while customers reporting headache were less likely to wait two or more days (OR = 0.567; 95 % CI 0.456-0.706) (see Table 3). Customers in the lowest wealth quintile (OR = 1.725; 95 % CI 1.146-2.595) and those diagnosed by a formally trained person were more likely to wait two or more days before visiting the PPMV shop (OR = 3.556; 95 % CI 2.213-5.715).

### Customer-PPMV attendant interactions

About 41 % of customers discussed the illness with the shop worker (see Table 2). After controlling for customer and PPMV shop worker characteristics in multivariate regression analysis, customers reporting fever or cough/cold symptoms were more likely to discuss the illness than if none of these symptoms were reported (OR = 2.738; 95 % CI 2.119-3.539 and OR = 1.688; 95 % CI 1.294-2.202, respectively) (Table 4, column 1). However, if the customer reported headache symptoms, they were less likely to discuss the illness with the PPMV worker (OR = 0.786; 95 % CI 0.626-0.988).

**Table 4:** Logistic regressions predicting customer-PPMV interactions (odds ratios)

	Discussed illness (1)	Sick person was examined (2)	Self/family/friend recommended drug (3)	Ln (cost)^a^ (4)	Had problems paying (5)
Symptoms reported					
Fever (ref: no fever)	2.738***	2.216***	0.353***	0.816***	2.404***
	[2.119 – 3.539]	[1.397 – 3.516]	[0.271 – 0.460]	[0.717 – 0.915]	[1.587 – 3.642]
Diarrhea (ref: no diarrhea)	1.351	0.605	0.739	−0.225***	0.990
	[0.935 - 1.951]	[0.251 - 1.462]	[0.514 - 1.062]	[-0.369 - -0.081]	[0.526 - 1.861]
Headache (ref: no headache)	0.786**	1.084	1.367***	−0.224***	0.753
	[0.626 - 0.988]	[0.684 - 1.718]	[1.096 - 1.706]	[-0.311 - -0.137]	[0.493 - 1.150]
Cough/cold (ref: no cough/cold)	1.688***	0.500**	0.686***	0.323***	1.969***
	[1.294 - 2.202]	[0.267 - 0.935]	[0.528 - 0.891]	[0.221 - 0.426]	[1.263 - 3.071]
Age (ref: 19-25)					
13-18	0.631**	0.420*	0.974	−0.281***	0.418**
	[0.441 – 0.903]	[0.169 – 1.044]	[0.699 – 1.355]	[-0.412 - -0.151]	[0.202 – 0.867]
26-40	1.282*	0.996	0.932	0.086	1.244
	[0.981 – 1.674]	[0.590 – 1.682]	[0.716 – 1.213]	[-0.017 – 0.189]	[0.772 – 2.007]
41+	1.367*	0.551*	0.906	0.135**	1.887**
	[0.989 - 1.891]	[0.291 - 1.044]	[0.658 - 1.248]	[0.009 - 0.261]	[1.072 - 3.324]
Education (ref: secondary)					
Primary	1.580***	2.231***	0.749**	−0.060	1.062
	[1.183 - 2.112]	[1.262 - 3.942]	[0.565 - 0.991]	[-0.170 - 0.050]	[0.652 - 1.730]
Higher	1.327*	1.248	0.972	0.063	0.488**
	[0.996 - 1.770]	[0.687 - 2.267]	[0.729 - 1.295]	[-0.048 - 0.175]	[0.274 - 0.867]
Wealth (ref: third quintile)					
Poorest	0.896	1.378	0.725	0.038	3.036***
	[0.598 - 1.342]	[0.620 - 3.061]	[0.490 - 1.074]	[-0.117 - 0.193]	[1.569 - 5.874]
Second	1.034	0.836	0.690**	0.026	1.667*
	[0.745 - 1.436]	[0.417 - 1.675]	[0.500 - 0.952]	[-0.099 - 0.151]	[0.949 - 2.929]
Fourth	1.119	0.871	0.690**	0.168***	0.777
	[0.813 - 1.540]	[0.451 - 1.682]	[0.505 - 0.943]	[0.047 - 0.290]	[0.415 - 1.455]
Richest	1.023	1.130	0.718**	0.279***	0.866
	[0.729 - 1.436]	[0.559 - 2.284]	[0.516 - 0.999]	[0.151 - 0.408]	[0.452 - 1.662]
Lives in community (ref: lives outside community)	0.948	0.819	0.805	−0.202***	1.145
	[0.695 - 1.294]	[0.452 - 1.486]	[0.591 - 1.096]	[-0.322 - -0.081]	[0.677 - 1.935]
Clinic near home (ref: no clinic near home)	1.047	1.430	0.988	0.056	1.021
	[0.826 - 1.327]	[0.860 - 2.378]	[0.786 - 1.243]	[-0.036 - 0.149]	[0.665 - 1.567]
Sick person age (ref: 18+)					
<5	0.890	0.452	0.855	0.455***	2.240**
	[0.579 – 1.368]	[0.175 – 1.166]	[0.554 – 1.321]	[0.293 – 0.617]	[1.194 – 4.202]
5-17	1.375*	0.839	0.823	0.210***	1.538
	[0.947 - 1.998]	[0.355 - 1.985]	[0.572 - 1.183]	[0.070 - 0.350]	[0.815 - 2.900]
Sick person is <18 and present at PPMV (ref: not present or 18+)	4.739***	23.400***	0.224***	0.323***	2.063**
	[2.885 - 7.785]	[9.799 - 55.883]	[0.131 - 0.383]	[0.152 - 0.495]	[1.094 - 3.891]
Sick person is male <18 (ref: female or 18+)	0.952	1.053	0.889	−0.125	0.557*
	[0.629 - 1.440]	[0.529 - 2.098]	[0.583 - 1.354]	[-0.277 - 0.027]	[0.309 - 1.004]
Diagnosed by formally trained person (ref: not diagnosed by formally trained person)	0.720*	1.627	0.072***	0.669***	1.466
	[0.492 - 1.053]	[0.844 - 3.139]	[0.040 - 0.127]	[0.523 - 0.815]	[0.806 - 2.668]
N	2155	2154	2155	2152	2120

ref = reference

***p < 0.01, ** *p* < 0.05, * *p* < 0.10

95 % confidence intervals in brackets. Standard errors are clustered at the shop level. All regressions control for customer age and tribe, PPMV age, gender, self-reported formal medical training, if PPMV offers tests/exams, state, and shop location (urban, peri-urban, or rural)

^a^Ordinary least squares analysis used

Children (age 13-18) purchasing drugs were significantly less likely to have a discussion about the illness with the shop worker than older customers aged 19-25 (OR = 0.631; 95 % CI 0.441-0.903) (see Table 4, column 1). Customers with primary education were more likely to discuss the illness compared to those with secondary schooling (OR = 1.580; 95 % CI 1.183-2.112). If the sick person was a child and s/he was present at the shop, the odds of discussing the illness was nearly five times higher than if s/he was not present or over age 18 (OR = 4.739; 95 % CI 2.885-7.785).

Even though less than one-quarter of PPMV shop workers interviewed reported having some formal medical training, over half (56.4 %) reported offering some sort of test or exam for customers (Table 1). An examination was observed in only 7.3 % of customer interactions (Table 2). In multivariate analysis, the odds of being examined were twice as high if fever was reported (OR = 2.216; 95 % CI 1.397-3.516) (Table 4, column 2). In contrast, customers reporting cough/cold symptoms were less likely to be examined (OR = 0.500; 95 % CI 0.267-0.935), and not significant association was detected for customers reporting headache symptoms. Sick individuals were more likely to be examined if they had only completed primary education (OR = 2.231; 95 % CI 1.262-3.942) or if s/he was a child present at the shop (OR = 23.400; 95 % CI 9.799-55.883).

During the exit interview, nearly half of customers (48.9 %) responded that they, another family member, or a friend rather than a doctor/nurse, pharmacist, CHEW, PPMV, or traditional healer recommended the drug purchased (Table 2). If fever or cough/cold symptoms were reported, the customer was less likely to select the drugs him/herself (OR = 0.353; 95 % CI 0.271-0.460 and OR = 0.686; 95 % CI 0.528-0.891, respectively); however, if headache was reported, the customer was more likely to do so (OR = 1.367; 95 % CI 1.096-1.706) (Table 4, column 3). Other characteristics associated with having a lower odds of self-recommending the drug included having primary education (OR = 0.749; 95 % CI 0.565-0.991), being in the second, fourth, or fifth wealth quintiles (compared to the middle quintile, OR = 0.690; 95 % CI 0.500-0.952, OR = 0.690; 95 % CI 0.505-0.943, and OR = 0.718; 95 % CI 0.516-0.999, respectively), being diagnosed by a formally trained person (OR = 0.072; 95 % CI 0.040-0.127), and purchasing for a sick child brought to the shop (OR = 0.224; 95 % CI 0.131-0.383).

In additional sensitivity analyses in multivariate regressions, we controlled for whether the PPMV worker was the shop owner or an apprentice; no results were different.

### Drug purchases

On average, customers spent 85.90 Naira (~US$0.52) on drug purchases, which could include vitamins/supplements and analgesics (Table 2). Different symptoms resulted in vastly different sums spent on drugs. Customers reporting fever spent an average of 203.71 Naira (~US$1.23), 111.37 Naira (~US$0.67) for those reporting cough/cold symptoms, 72.49 Naira (~US$0.44) for diarrhea, and 69.11 Naira (~US$0.42) headache symptoms. In multivariate analysis, fever was associated with spending 81.6 % more than if no fever was reported (95 % CI 0.717-0.915), and those experiencing cough/cold symptoms spent nearly one-third more than if cough/cold was not experienced ($$ \widehat{\widehat{\beta}} $$ = 0.323; 95% CI 0.221-0.426) (Table 4, column 4). Conversely, customers purchasing for diarrhea or headache spent 22 % less than customers without those symptoms (95 % CI -0.369- -0.081 and -0.311- -0.137, respectively).

Customers aged 13-18 spent 28.1 % less compared to those age 19-25 (95 % CI -0.412- -0.151), while those over age 40 spent 13.5 % more (95 % CI 0.009-0.261). Customers in the two richest wealth quintiles spent 16.8 % and 27.9 % more than customers in the middle wealth quintile (95 % CI 0.047-0.290 and 0.151-0.408, respectively), and if the customer lived in the same community as the shop’s location, s/he spent 20.2 % less compared to those living elsewhere (95 % CI -0.322- -0.081). More was spent if the sick person was a child: 45.5 % more if the sick person was under age five (95 % CI 0.293-0.617) and 21.0 % more if s/he was age 5-17 (95 % CI 0.070-0.350). When the sick child was brought to the shop, 32.3 % more was spent (95 % CI 0.152-0.495). Being diagnosed by a formally trained person before attending the shop was also associated with spending 66.9 % more (95 % CI 0.523-0.815).

About 10 % of customers reported difficulty paying for their drug purchases (see Table 2). Symptoms associated with a higher likelihood of payment difficulties included fever (OR = 2.404; 95 % CI 1.587-3.642) and cough/cold (OR = 1.969; 95 % CI 1.263-3.071), but not diarrhea or headache (Table 4, column 5). Customers aged 13-18 were less likely to have problems paying (OR = 0.418; 95 % CI 0.202-0.867) while those over age 40 were more likely (OR = 1.887; 95 % CI 1.072-3.324). Fewer problems paying was associated with higher education (OR = 0.488; 95 % CI 0.274-0.867), but greater difficulty paying was associated with being in the poorest wealth quintile (OR = 3.036; 95 % CI 1.569-5.874), for caregivers purchasing for a sick child under age five (OR = 2.240; 95 % CI 1.194-4.202), and if the sick child was brought to the shop (OR = 2.063; 95 % CI 1.094-3.891).

**Fig. 1:**
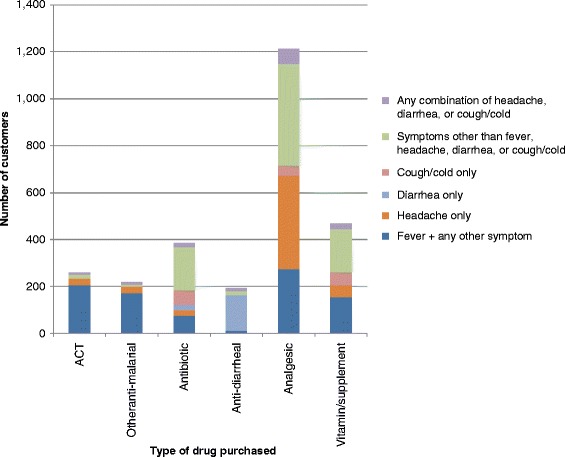
Drug type purchased by reported symptom. The number of customers reporting different symptoms was plotted according to which type of drug was purchased. Some customers purchased more than one drug type. Fever was often reported with other symptoms (such as headache, cough/cold, or others) and all combinations of fever with other symptoms were grouped together. ACT = artemisinin-based combination therapy

Finally, the type of drugs purchased for different reported symptoms was described (Fig. 1). Analgesics were purchased in greatest quantity to treat a wide variety of symptoms, including headache, fever, cough/cold, or other symptoms such as sweating, nausea/vomiting, body aches/joint pain, or chills/shivering. Although fewer of them were purchased, vitamins/supplements and antibiotics were also used to treat a wide variety of ailments. Most customers (82 %) who purchased an ACT or other anti-malarial reported fever symptoms and 82 % of customers purchasing an anti-diarrhea product reported diarrhea symptoms.

## Discussion

Based on PPMV shop and customer surveys and observations in Kogi and Kwara states, Nigeria, the major symptoms for which customers (adults and children) seek care at PPMV shops were fever, cough/cold, and diarrhea. The prevalence of these symptoms was similar to what has been previously reported among children at health facilities in Nigeria [33–35]. However, this is the first study to document that a similar prevalence of these symptoms exists among adults seeking care at PPMV shops.

Studies of individual care-seeking in many LMICs suggests a complicated process according to a number of factors, including symptom [4, 33, 36], consultation with household members [37–39], relying on prior experiences [40], perceived severity of illness [41], quality of care, provider reputation [42, 43], cost [7, 38], location [44], and use of multiple, concurrent treatments [4, 45, 46]. Our study of care-seeking showed that PPMV customer behavior also varied by many of these factors. That customers with fever were more likely to get diagnosed by a formally trained person, discuss their symptoms with and be examined by the shop worker, and less likely to self-recommend purchased drugs suggests that customers may perceive fever to be a more serious symptom of illness than the other symptoms. In contrast, customers with cough/cold or headache symptoms were more likely to seek care in a way consistent with self-treatment (i.e. less likely to be diagnosed by a formally trained person and discuss the illness with the shop worker or be examined, and more likely to select the drug themselves) rather than consulting a provider.

Differences in care-seeking behavior were also observed across wealth strata, which suggest that there are substantial equity gaps in access to care, even among individuals who seek care at informal providers. Customers in the two richest wealth quintiles were more than twice as likely to have sought care from a formally trained provider before going to a PPMV shop compared to customers in lower quintiles, with those in the poorest quintile more likely to wait two or more days before seeking care. Previous studies also show that poorer households in Nigeria are more likely to use informal private providers [47] while richer households are more likely to go to private clinics [48–50]. These differences by wealth are likely be related to cost of care, which has also been found to cause poorer households to delay care-seeking, at both formal facilities and informal drug sellers [34, 40]. Evidence from this and other studies [44] also indicates that distance can be a barrier to care-seeking: customers living near a clinic were slightly more likely to have been diagnosed by a formally trained provider.

While most of the care-seeking and customer-provider interactions outcomes analyzed were similar for adults and children, delayed care-seeking and improper diagnosis and treatment have more severe implications for children given their high risk of mortality from common illnesses. Only 8 % of customers captured in this study were diagnosed by a formally trained person before visiting the PPMV shop, akin to what other studies have found [22, 34, 51]. Furthermore, when caregivers face financial and distance-related barriers, and have difficulty recognizing different illnesses or their severity, they are more likely to try home or multiple treatments in an effort to “buy time” while they monitor disease progression [4, 19], delaying proper treatment [16, 40, 41, 51]. However, studies show that households may be more willing to pay for higher quality drugs for children than adults [43, 51], which was also the case in this study. When seeking care for children, mothers have ranked cost as less important than when seeking care for themselves [34]. Moreover, if the sick child was brought to the PPMV shop, a discussion of the current illness episode and an exam was much more likely to occur, and caregivers where less likely to rely on their own recommendation for purchasing drug. This finding corroborates other studies in Nigeria which show that children are more likely to receive “better” care than adults, although improper treatment may still be high [51, 52] and may oftentimes be irrational and lead to excessive drug expenses [24]. It was difficult to discern any systematic patterns in drug purchases for different symptoms. The large volume of analgesic and vitamin/supplement sales accords with the regulatory scope of PPMVs as over-the-counter drug sellers. Yet, customers tended to spend more when purchasing drugs to treat fever and cough/cold symptoms. Further, while most fevers were associated with the purchase of anti-malarial drugs, not all were artemisinin-based combination therapies (ACTs) recommended by national treatment guidelines [49]. And since PPMVs are not allowed to perform diagnostics [17, 53], whether febrile and non-febrile illnesses were treated correctly cannot be determined [51, 54]. As such, many antibiotics were also purchased even though PPMVs are not legally permitted to sell them, highlighting the influence of both strong consumer demand for these drugs and weak regulatory enforcement on PPMV dispensing. Overall, the lack of consistent dispensing among PPMVs may be partly attributable to the fact that PPMVs are not required to undergo any formal pharmacy training, and many may not have adequate knowledge of primary care for the common illnesses they are presented with [55].

The findings from this study should be noted with several limitations. Recruitment occurred at PPMV shops that were part of a targeted, stratified random sample, which may not be representative of all retail shops in the area. Only shops in two states were used for recruitment, and PPMV shops in other states may be used differently. Care-seeking behavior in North Central Nigeria may also not be representative of health behaviors in other parts of the country. Most responses were self-reported and there may be recall bias or other important mediating factors, such as household decision-making dynamics over healthcare expenditures, which influence care-seeking behaviors that were not observed. Although surveyors did not engage with either the shop worker or customer during the transaction, his/her presence alone may have influence either to behave in a more socially desirable manner, which may result in more favorable responses. The customer survey did not collect information on treatment attempted before attending the PPMV shop, so this aspect cannot be evaluated. Finally, no formal diagnosis was available for these customers so it was not possible to assess how care-seeking behavior changes based on actual illness and not just symptoms. Without a diagnosis, the quality of care received at the PPMV shop could not be evaluated, since receiving proper treatment could not be measured. Although compensation gifts were not given to the shop worker until the end of the survey day to minimize response or behavior bias, we cannot rule out the possibility that providers may have behaved differently because of any perceived benefits of participation.

## Conclusions

A variety of previous research on PPMV practice in Nigeria has noted the generally poor quality of health services delivered by these providers [25]. While improper care-seeking may contribute to the lack of appropriate care, our research also shows that care-seeking behavior depended on the symptoms experienced and the sick person’s age, with fever symptoms and young children given relatively more attention in terms of diagnosis, consultation, and speed. Other symptoms—diarrhea, cough/cold, and headaches—received relatively less attention, including circumventing formal diagnosis and delayed care-seeking, which may exacerbate more severe underlying illnesses, such pneumonia, which also significantly contribute to child mortality in Nigeria [56].

Interventions to improve primary care should develop basic guidelines for common illnesses, for both adults and children. Caregivers of children with fever, diarrhea, and headache symptoms should be seeking care promptly given that the leading causes of death among children in Nigeria stem from common preventable illnesses—diarrhea, pneumonia, and malaria [56]. Given that caregivers are often unable to recognize different illnesses, interventions aimed at improving health outcomes for children should target caregiver behavior change and increase demand for diagnosis from a formally trained medical provider before treatment. This includes bringing the sick child to the shop in order to increase the likelihood of a more thorough consultation, provided that the PPMV worker is adequately trained to identify and assess childhood illnesses.

While not necessarily viewed as formal medical providers, training PPMVs to conduct some basic health consultations, along with strong referral skills, may help to provide better care for lower SES populations and those living farther away from a health facility who are more likely to use PPMVs in lieu of formal medical care. Complementary approaches, that not only focus on formal care through the existing primary care system, but also on community-based, informal sources of care, may help to achieve universal health coverage goals. However, rationalization of current policies and treatment guidelines is needed to improve quality of care and dispensing at PPMVs, including setting and enforcing standards with respect to antibiotics, basic diagnostic tools, and training that could help to guide drug selling practices.

## Abbreviations

ACT: Artemisinin-based combination therapy
CHEW: Community health extension worker
PPMV: Patent and proprietary medicine vendor
RDT: Rapid diagnostic test
